# Regeneration or Repair? The Role of Alveolar Epithelial Cells in the Pathogenesis of Idiopathic Pulmonary Fibrosis (IPF)

**DOI:** 10.3390/cells11132095

**Published:** 2022-06-30

**Authors:** Paola Confalonieri, Maria Concetta Volpe, Justin Jacob, Serena Maiocchi, Francesco Salton, Barbara Ruaro, Marco Confalonieri, Luca Braga

**Affiliations:** 1 Pulmonology Department, Cattinara Hospital, University of Trieste, 34127 Trieste, Italy; paola.confalonieri@asugi.sanita.fvg.it (P.C.); francesco.salton@asugi.sanita.fvg.it (F.S.); barbara.ruaro@units.it (B.R.); marco.confalonieri@asugi.sanita.fvg.it (M.C.); 2 Department of Medical Surgical and Health Sciences, University of Trieste, 34127 Trieste, Italy; 3 Functional Cell Biology Group, International Centre for Genetic Engineering and Biotechnology (ICGEB), 34149 Trieste, Italy; volpe@icgeb.org (M.C.V.); justin.jacob@icgeb.org (J.J.); serena.maiocchi@icgeb.org (S.M.); 4 Life Sciences Department, University of Trieste, 34128 Trieste, Italy

**Keywords:** IPF, ATII cells, regeneration, senescence

## Abstract

Idiopathic pulmonary fibrosis (IPF) is a chronic, progressive interstitial lung disease (ILD) with unknown etiology in which gradual fibrotic scarring of the lungs leads to usual interstitial pneumonia (UIP) and, ultimately, to death. IPF affects three million people worldwide, and the only currently available treatments include the antifibrotic drugs nintedanib and pirfenidone, which effectively reduce fibrosis progression are, unfortunately, not effective in curing the disease. In recent years, the paradigm of IPF pathogenesis has shifted from a fibroblast-driven disease to an epithelium-driven disease, wherein, upon recurrent microinjuries, dysfunctional alveolar type II epithelial cells (ATII) are not only unable to sustain physiological lung regeneration but also promote aberrant epithelial–mesenchymal crosstalk. This creates a drift towards fibrosis rather than regeneration. In the context of this review article, we discuss the most relevant mechanisms involved in IPF pathogenesis with a specific focus on the role of dysfunctional ATII cells in promoting disease progression. In particular, we summarize the main causes of ATII cell dysfunction, such as aging, environmental factors, and genetic determinants. Next, we describe the known mechanisms of physiological lung regeneration by drawing a parallel between embryonic lung development and the known pathways involved in ATII-driven alveolar re-epithelization after injury. Finally, we review the most relevant interventional clinical trials performed in the last 20 years with the aim of underlining the urgency of developing new therapies against IPF that are not only aimed at reducing disease progression by hampering ECM deposition but also boost the physiological processes of ATII-driven alveolar regeneration.

## 1. Idiopathic Pulmonary Fibrosis

### 1.1. Definition and Epidemiology

Diffuse parenchymal lung diseases, often collectively referred to as interstitial lung diseases (ILDs), belong to a heterogeneous group of disorders that are classified together because they share similar clinical, radiographic, physiologic, or pathologic manifestations [1]. All ILDs present with interstitial thickening and/or fibrosis besides extensive yet variable alterations of alveolar and airway architecture, usually leading to decreased lung volume and compliance and eventually to inadequate oxygenation due to both impaired ventilation and impaired gas exchange [2]. 

Idiopathic pulmonary fibrosis (IPF) accounts for 20% of all ILDs in clinical practice, ranging from 0.9 to 14 cases/100.000/y in incidence worldwide [3,4]. IPF is a chronic, progressive ILD characterized by usual interstitial pneumonia (UIP) pattern, defined through histology or through high-resolution computed tomography scan (HRCT) in the absence of an evident etiology. The progressive lung scarring that occurs in IPF is burdened by a high mortality associated with respiratory failure, as median survival does not exceed 5–10 years from diagnosis in most cases, despite the availability of antifibrotic drugs [2]. IPF also represents an economic burden, with a direct treatment cost of around USD 25,000/person-year, which is a higher cost in comparison to breast cancer and many other life-threatening medical conditions [5].

**Figure 1:**
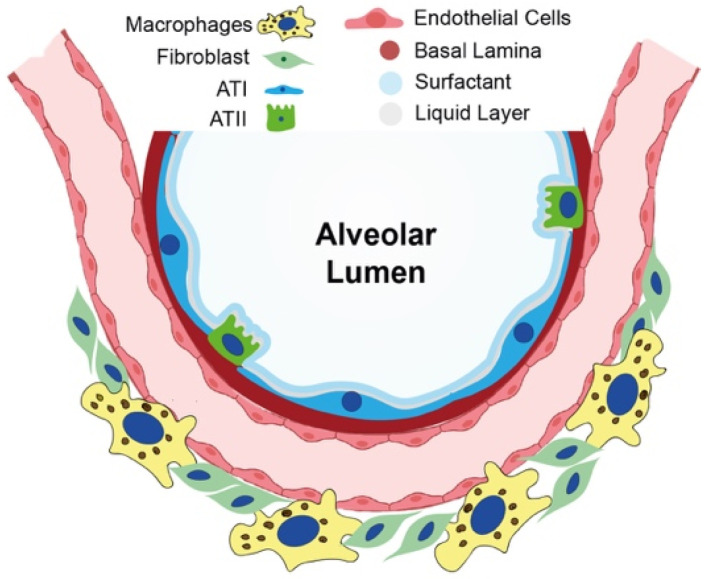
Cell composition of the healthy alveolus. Schematical image of the alveolar compartment: the alveolar lumen (white) is surrounded by ATI cells (blue) and ATII cells (green), which secrete surfactant (light blue). The alveolar epithelial basal lamina (pink) separates alveolar epithelial cells from alveolar endothelial cells. Pulmonary interstitial tissue is schematically represented as a compartment mainly composed of fibroblasts (light green) and macrophages (yellow), which are known to be the most relevant interstitial cell types in the pathogenesis of IPF.

The biological processes involved in IPF onset are still a matter of considerable interest for the scientific community; however, a common consensus has been established in considering repetitive alveolar epithelial injury followed by an aberrant regenerative response as the main trigger [6]. Alveoli are lined by a continuous epithelium composed of intermixed alveolar epithelial type I (ATI) and alveolar epithelial type II (ATII) cells, and they are separated from one another by interalveolar septa. ATI cells, which represent around 40% of the alveolar cellular population and cover 90% of the alveolar surface area, are differentiated cells with a thin and flat shape with multiple branches spread over a large area of the alveolar epithelial basal lamina, facilitating contact with alveolar endothelial cells and promoting gas exchange. Instead, ATII cells, which account for about 60% of the alveolar cells and cover the remaining 10% of the alveolar surface area, are small, cuboidal cells. Their primary function is to reduce surface tension by the secretion of surfactant, mainly composed of phospholipids, such as dipalmitoylphosphatidylcholine; unsaturated phosphatidylcholine and neutral lipids; and surfactant-associated proteins, termed surfactant proteins C (SPC), B (SPB), A (SPA), and D (SPD). SPC and SPB are essential proteins for preventing alveolar collapse during the respiratory act [7]. In addition, ATII cells regulate innate immunity in the lung via secretion of collectins; SPA and SPD; and anti-inflammatory, antimicrobial mediators, such as lysozymes and defensins [7] (Figure 1). Defects of pulmonary surfactants in infants are linked with respiratory distress syndrome (ARDS) or, in adults, with acute ARDS and interstitial lung diseases [8].

Upon repetitive alveolar epithelial injury, IPF susceptibility is mainly determined by genetic and environmental determinants [6]. In particular, cigarette smoking represents the highest risk factor, followed by conditions linked to occupational exposure, such as agriculture and farming, livestock, wood dust, metal dust, stone dust, and silica [9,10,11]. Susceptibility is also determined by the genetic background; in the past decade, thanks to substantial advances in genomics and increased accessibility to whole-exome sequencing technology, several large genome-wide association studies have identified common genetic variants that appear to increase the risk of developing IPF. The most common polymorphism associated with IPF is the rs35705950 variant of the mucin 5b (MUC5B) gene promoter [12,13]. Mucins are a family of high-molecular-weight, heavily glycosylated proteins involved in mucus production by lung epithelial tissues [13]. Moreover, small family-based genetic studies allowed the identification of rare IPF-associated genetic mutations (familial IPF) that are mainly related to telomere maintenance (cellular senescence) and surfactant production and secretion. Multiple genes related to telomere maintenance, such as TERT, TERC, TINF2, DKC1, RTEL1, PARN, and NAF1, have been found to be mutated in 25% of patients with familial IPF (F-IPF) [13]. It is believed that telomere shortening and resulting cell senescence might be one of the causes of loss of regenerative capacity after injury in ATII cells [14]. Similarly, mutations in genes involved in surfactant protein production and secretion (SFTPC, SFTPB, SFTPA, and ABCA3) are known to be causative of dysfunctional folding, processing, and secretion of surfactant and therefore to induce ATII toxicity and, eventually, promote the loss of functional ATII cells [15,16]. Due to the considerable overlap of the genetic landscape in sporadic and familial IPF, a sharp classification in these two categories appears complicated and these pathological conditions are most likely associated with a continuum of genetic risk for pulmonary fibrosis rather than two distinct syndromes [17]. 

It would be fascinating to elucidate how a mutation in ATII-specific genes, such as SFTPC and ABCA3, leads to clinical manifestation of sporadic IPF. This would shed light on whether IPF can be considered an epithelium-driven disease, as well as on the importance of mutated genes in the development of effective therapies. Furthermore, it is imperative to better understand whether epithelial damage alone, without exogenous injury, can trigger the initiation of the fibrotic response in the lungs.

### 1.2. Clinical Manifestation and Diagnosis

Typically, patients with IPF present with progressive exertional dyspnea, dry cough, and exercise limitations [18]. These symptoms are initially mild and nonspecific and are usually attributed to aging or other pulmonary and cardiovascular conditions, making early diagnosis of IPF challenging. Patients with IPF rarely present with an acute exacerbation—an unexplained worsening of dyspnea over a few weeks and, eventually, flu-like symptoms—as an initial manifestation [19].

IPF is a typical disease of older age, rarely occurring before the fourth decade of life. On the contrary, F-IPF differs in terms of disease onset, occurring at an early age. Notably, in F-IPF, the penetrance appears to vary substantially, resulting in a clinical onset of symptoms between 4 months from birth to more than 60 years of age, supporting the “two-hit” concept with respect to the development of the disease: on one hand, a genetic predisposition, resulting in epithelial injury; and on the other hand, environmental hits, including cigarette smoke and exposure to outdoor pollution [20].

Upon physical examination, bibasilar inspiratory Velcro-like crackles are nearly universally audible by chest auscultation, whereas digital clubbing and acrocyanosis are common manifestations of advanced IPF [19]. Upon testing of pulmonary function, a reduced diffusion lung capacity for carbon monoxide is already detectable in the early stage of IPF, followed by the appearance of a restrictive ventilator defect on spirometry and, eventually, reduced total lung capacity [21]. The 6 min walking test may detect desaturation upon exertion, and it has prognostic value, as faster decline in distance covered is associated with higher mortality [22].

When history and physical examination suggest interstitial lung disease, a high-resolution CT scan of the chest should be obtained to determine whether the disease is present and to identify specific radiologic patterns [23]. A thorough history, including environmental exposures, medication use, and extrapulmonary symptoms, is fundamental to rule out identifiable causes of ILDs, such as domestic and occupational exposure, connective tissue disease, and drug toxicity.

Indeed, the diagnosis of IPF is based on the typical radiological and histological appearance of usual interstitial pneumonia (UIP) without an apparent underlying cause. The radiologic pattern of UIP consists of bilateral reticulation and honeycombing that is predominantly peripheral and in the lower lobes, with a correspondent histologic UIP pattern consisting of heterogeneous paraseptal fibrosis with architectural distortion associated with the typical presence of fibroblast foci [19,24]. The guideline on IPF diagnosis published in 2018 by ATS/ERS/JRS/ALAT defines three diagnostic categories based on the pattern observed with HRCT: UIP definite, probable UIP, and indeterminate for UIP [19]. In the case of HRCT results showing a canonical UIP definite pattern, HRCT is sufficient to conclude a diagnosis of UIP/IPF without the need for more invasive testing or histology [19]. In the case of an uncertain HRCT pattern, the suggested procedure to confirm the diagnosis is video-assisted thoracoscopic wedge biopsy (VATS) [19].

Considering the challenge in achieving a correct diagnosis, multidisciplinary discussion (MDD) among different experts (including clinicians, radiologists, pathologists, and rheumatologists) is recommended by the international guidelines because it has been demonstrated to improve diagnostic accuracy and interobserver agreement [23].

## 2. Current Knowledge of Cellular and Molecular Mechanisms in IPF Onset

### 2.1. Epithelial-to-Mesenchymal Transition as a Nonmesenchymal Source of Fibrosis 

In fibrotic conditions, epithelial-to-mesenchymal transition (EMT) occurs in ATII cells [25] adjacent to sites of extracellular matrix (ECM) deposition, setting a profibrogenic microenvironment due to the loss of the epithelial phenotype, the acquisition of a mesenchymal phenotype, and the activation of fibroblasts into myofibroblasts, eventually causing the development of fibrosis [26].

EMT is a biological process in which epithelial cells lose contact adhesion and apical–basal polarity, acquiring mesenchymal features, such as invasion, migration, and secretion of extracellular matrix components [27]. EMT is also an essential process during embryonic development, gastrulation, and development of the neural crest, lungs, heart, and other organs [28]. However, it can also occur as a response to injury and carcinogenesis, as well as in fibrosis. Because EMT is involved in so many biological processes, it has been classified into three different types as follows: (1) type I is associated with physiological processes involved in tissue and organ formation during embryogenesis; (2) type II refers to normal wound healing and has a role in excessive tissue repair; finally, (3) type III involves the acquisition of a migratory phenotype by malignant epithelial cells and is associated with tumor invasiveness and metastasis. EMT in IPF has long been categorized as type II. It is physiologically induced in response to injury and stops when tissue repair leads to wound healing and subsequent regeneration [29]. It has been observed that EMT contributes to the early development of interstitial fibrosis via paracrine signaling directed from the alveolar epithelium to underlying fibroblasts, inducing their activation into myofibroblasts [26]. EMT is also a progressive event in which epithelial cells undergo different changes, such as modifications in transcriptional regulation (loss of positivity for E-cadherin and cytokeratins, as well as expression of ZEB-1, ZEB-2, Twist, Vimentin, and β-catenin, described as the main key regulators of EMT) as well as variations in cytoskeleton and motility, in cell adhesion, and in the production of ECM components. EMT of ATII cells is mainly sustained by the aberrant activation of specific EMT-related pathways, such as the Wnt, Sonic Hedgehog (SHH), and TGF-β signaling pathways. These pathways crosstalk with each other and converge to the key transcription factors, SNAIL-1, SNAIL-2, ZEB-1, and ZEB-2, inducers of EMT and inhibitors of the E-cadherin promoter, to initialize and maintain the process of EMT [30]. Especially in an adult lung during the EMT process, Wnt signaling promotes induction and stabilization of β-catenin. Wnt activates the SMAD pathway, contributing to the cytosolic accumulation of β-catenin and translocation of factors into the nucleus, causing modifications in cytokeratin expression and reorganization of the cytoskeleton [27]. Another of the most studied inducers of EMT is the growth factor TGF-β. It activates SMAD2/3, which, in turn, forms a complex with SMAD4 and participates directly in the transcriptional regulation of SNAIL-1 and SNAIL-2. Finally, the SHH pathway contributes to sustenance of the EMT process on different levels, promoting transcriptional changes that lead to loss of the adherent junction complex, breakdown of the apical–basal polarity, and cytoskeleton rearrangement [30]. However, the role of EMT in the pathogenesis of IPF is still the subject of debate. In 2011, Rock and colleagues demonstrated that there is no evidence that ATII cells can turn into stromal cells using a C-CreER^T2^ knock-in allele to mark ATII cells in vivo. On the contrary, they proved that ATII cells transdifferentiated into ATI cells upon bleomycin-induced lung fibrosis [31].

**Figure 2:**
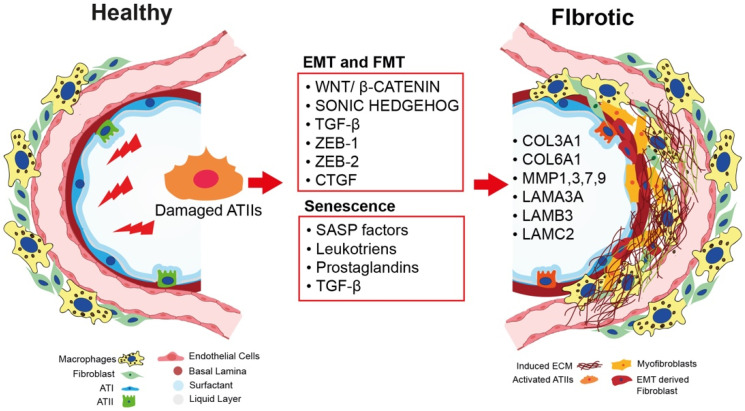
Key processes inducing pulmonary fibrosis. Repetitive injuries in human adult lung lead to damaged ATII cells (orange). Damaged ATII cells begin overexpressing the Wnt/β-catenin and SHH signaling pathways and secreting TGF-β. They also start expressing ZEB-1, ZEB-2, and CTGF as a response to TGF-β secretion. These factors are involved in the EMT and FMT processes. In parallel, damaged ATII cells acquire a senescent phenotype, producing SASP factors, leukotrienes, and prostaglandins. These factors collectively contribute to the proliferation of myofibroblasts which, in turn, are responsible for the aberrant deposition of ECM components, such as COL3A1, COL6A1, MMP1, MMP3, MMP7, MMP9, LAMA3, LAMB3, and LAMC2.

Pulmonary fibrosis is also an age-related disease, with a median age of diagnosis of 66 years old [32]. As such, several factors that accumulate with age have been found to contribute to ATII cell dysfunction, which, in turn, leads to the development of lung fibrosis. Senescent ATII cells secrete high levels of growth factors, cytokines, and chemokines, which promote abnormal myofibroblast differentiation and persistent tissue remodeling [33]. Recently, Moimas et al. reported that ATII cells purified from IPF patients exhibit high levels of the p21 and p16 senescence markers, as well as the ZEB-1 and ZEB-2 EMT markers, demonstrating that these two events cross react with each other [25]. Another work published by Muthuramalingam [34] revealed that the A549 human cell line treated with bleomycin in vitro expressed high levels of senescence and EMT markers, favoring a pro-fibrotic microenvironment that could lead to secretion of TGF-β, identified as the central mediator that links EMT and senescence [34]. Moreover, Faheem et al. showed that in carcinogenesis, senescent cells secrete high levels of senescence-associated secretory phenotype (SASP) factors and TGF-β, promoting the activation of the EMT process and, later, leading to the myofibroblastic differentiation and production of ECM components [35]. Further studies are needed to understand the link between EMT and senescence in lung fibrosis [36] (Figure 2). 

### 2.2. The Role of Bidirectional Epithelial–Mesenchymal Crosstalk in Fibroblast-to-Myofibroblast Activation

For years, IPF was considered a devastating disease in which inflammatory or mesenchymal cells represented the main drivers of IPF due to the high amount of inflammation and the consistent amount of collagen deposition in the lungs [37]. 

However, in the recent years, evidence has shown that IPF is an epithelium-driven disease, indicating that ATII cells could play a pivotal role in the pathological activation of fibroblasts and, therefore, in the pathogenesis of IPF [38]. Pathological fibrogenesis involves complex crosstalk between alveolar epithelial cells, fibroblasts, immune cells, and endothelial cells [39,40]. IPF lungs show an aberrantly activated lung epithelium in which dysfunctional ATII cells may produce mediators of fibroblast migration, as well as release TGF-β and recruit inflammatory cells, leading to fibroblast proliferation and activation into myofibroblasts [41]. The origin of pathological myofibroblasts is still poorly understood. The majority of the scientific community considers EMT as a potential source of fibrosis, leading to the transformation of epithelial cells into ECM-secreting myofibroblasts [42,43]. Recently, Yao et al. identified a novel regulatory axis where EMT is involved in the development of fibrotic lesions via paracrine activation of fibroblasts [26]. They showed that the epidermal growth factor receptor (EGFR)-RAS-extracellular signal-regulated kinase (ERK) signaling pathway activates the transcription factor ZEB1, which not only is involved in the EMT event but also controls the production of mediators, contributing to fibrosis via epithelial–fibroblast crosstalk. Most recently, Yao et al. also showed that this event does not occur only in ATII cells first and in fibroblasts later but also in the reverse order, producing reciprocal paracrine signaling. They showed that both TGF-β-activated fibroblasts and IPF fibroblasts induce RAS activation in ATII cells, and this process is at least partially driven by the secreted protein acidic and rich in cysteine (SPARC), supporting the concept that aberrant bidirectional epithelial–mesenchymal crosstalk contributes to the development of a profibrogenic microenvironment, where a chronic wound-healing response leads to the development of lung fibrosis [44].

Although much of the pathogenesis of IPF remains to be elucidated, fibroblasts and ATII cells have emerged as principal players in this disease. The mechanism by which exhausted ATII cells can activate fibroblastic differentiation into myofibroblasts has been an area of controversy. Many published papers have shown that ATII cells purified from IPF patients are senescent and apoptotic [25,45,46]. Interestingly, in normal lungs, ATII cells are physically separated from the fibroblasts by the alveolar epithelial basal lamina, although it has been shown that in IPF, areas of apoptotic ATII cells and foci of alpha-smooth muscle actin (α-SMA)-positive myofibroblasts colocalize [47], suggesting that these two types of cells influence each other as fibrosis develops. On the other hand, ATII cells are capable of activating fibroblasts by releasing factors such as TGF-β; cytokines; growth factors, such as the connective tissue growth factor (CTGF); and morphogens, such as sonic hedgehog (SHH) [45], which are the best-known factors studied in in vitro co-culture experiments. There are also lipid mediators, such as prostaglandin E2 (PGE2), known to be an antisuppressive player in the activation of fibroblasts through paracrine stimuli (Figure 2).

### 2.3. Cellular Senescence and IPF: A Causative Link 

Because IPF is an age-related disorder and 25% of familial IPF cases are associated with mutations affecting telomere maintenance [13], it appears evident that cellular senescence plays a relevant role in the development of this chronic disease [48]. Aging processes have been shown to push ATII cells to acquire a specific phenotype that is characterized by replicative arrest and the aberrant secretion of profibrotic and proinflammatory senescence-associated factors (metalloproteinases (MMPs), TNFα, CCL2, CXCL1, TGF-β, IL-6, IL-8, CCL2, PAI-1, IGFBP-2, and leukotrienes (LTs)) [49,50,51,52,53]. This phenotype is called senescence-associated secretory phenotype (SASP). 

In Humans, it is known that senescent cells (β-Gal- and p16-positive) with evident SASP accumulate with age in adult lungs; these cells exert autocrine and paracrine effects, resulting in increased lung dysfunction and/or senescence of neighboring cells [50,51,54]. Accordingly, the presence of senescent ATII cells (p16- and p21-positive) has been confirmed in IPF lungs by single-cell RNA sequencing experiments [55,56,57,58].

In addition, fibrotic remodeling during advancing age and cellular senescence not only impairs the regenerative function but also affects the surfactant function of ATII cells [59,60]. The order of events occurring during the pathogenesis of IPF has not been elucidated yet, but it is known that the collapse of the alveolus occurs due to the alteration of its surfactant system. Autosomal dominant mutations in the surfactant protein C, such as the variant SFTPC-I73T, are expressed exclusively in ATII cells and are known to F-IPF [61]. A previous study conducted with a rat model of bleomycin-induced fibrosis confirmed downregulation of SP-B and SP-C gene expression [62]. Likewise, Rodriguez et al. attributed this downregulation of surfactant proteins to the activation of TGF-β1 signaling [63]. Yazicioglu and his group correlated age-related changes with impaired surfactant metabolism and elevated inflammation in the ATII cells isolated from young and old mice upon induction of acute lung injury. Notably, an increase in the expression of DNA damage markers, as well as regulator of cell cycle arrest Trp53, was observed [64].

Leukotrienes (LTs) and prostaglandins (PGLs) are known to be implicated in the pathophysiology of pulmonary fibrosis, and their molecular role has been extensively investigated. It has been shown that LTs are also produced by senescent SASP-positive cells and that the senolytic agent AB-236 suppresses fibrosis, reducing the abundance of LTs in broncho alveolar lavage fluid (BAL-F) sampled in a mouse model of bleomycin-induced pulmonary fibrosis [52].

The first comprehensive proteomic analysis of SASP factors originating from multiple senescence stimuli in different cell types was published in 2020 [65]. In particular, the secretome of human lung fibroblasts (IMR90) challenged with different pro-senescence stimuli (X-ray irradiation (IR), inducible RAS overexpression (RAS), or ATZ stimulation) was analyzed by mass spectrometry, and this unbiased profiling identified numerous secreted proteins as increased in senescent fibroblasts versus control cells. Besides known SASP factors (CXCLs, HMGB1, IGFBPs, and MMPs) unique to each specific treatment, additional clustering analysis identified CXCL1, MMP1, STC1, PAI-1, and GDF15 as a subset of SASP proteins that are common to all senescence triggers [65].

Interestingly, MMP1, MMP7, CCL2, CXCL1, IGFBP-2, and PAI-1 are secreted factors that have been found to be increased in patients with progressive fibrosis, as well as to be secreted by cells with evident SASP, suggesting a close mechanistic connection between IPF and cellular senescence [50,51,54] (Figure 2).

Exploiting an in vitro model of bleomycin-induced epithelial cell senescence, the supernatant of senescent lung epithelial cells was able to mediate fibroblast-to-myofibroblast transition (FMT) and pro-fibrotic responses in pulmonary fibroblasts [50,51]. Similarly, the supernatant of senescent lung fibroblast is sufficient to trigger FMT in non-senescent fibroblasts at a level comparable to that induced by TGF-β treatment [51]. 

Myofibroblasts are generally resistant to apoptosis and highly proliferative, causing a massive deposition of highly fibrillar ECM components (fibrillar collagens, fibronectin, tenascin, and proteoglycans) in the lung interstitial space [66]. 

Overall, it is clear that, independent of the triggering event, the SASP of both ATII cells and fibroblasts is crucial in the initiation and development of progressive pulmonary fibrosis. Recently, Yao et al. generated a novel mouse model of conditional ATII cell senescence without loss of ATII cells. In particular, the ATII-restricted silencing of Sin3a led to impaired progenitor cell function and induction of p53-dependent ATII senescence, resulting in spontaneous progressive pulmonary fibrosis [67].

In 2016, Hashimoto et al. provided striking evidence that cellular senescence may be a relevant target for lung functional improvement [68]. They discovered that the elimination of naturally occurring senescent cells by a novel in vivo suicide gene strategy completely restores lung function in aged mice [68]. Accordingly, a recent study corroborated this evidence in humans, confirming the capacity of senolytic drugs to reduce the amount of collagen deposition and alleviate lung dysfunction in a larger randomized clinical trial [69]. In conclusion, cellular senescence and chronic SASP are key regulators of pathological lung remodeling, including all age-related lung pathologies and lung diseases that are characterized by stress-induced premature senescence.

### 2.4. The Role of ECM in IPF Onset and Progression

As described above, cellular senescence plays a role in IPF lungs, but the mechanisms by which the senescence of ATII cells leads to IPF onset and progression have not been fully elucidated yet. As mentioned in the previous paragraph, senescent ATII cells acquire a phenotype called SASP, which is characterized by the secretion of proteins that affect nearby cells by promoting the activation of fibroblasts [44]. Among SASP factors, there are metalloproteinases (MMPs), such as MMP2 and MMP9, that belong to a family of zinc-dependent endopeptidases essential for ECM degradation and remodeling [49,70,71].

During the normal respiratory cycle, the airway epithelium stretches and relaxes, and these dynamics are exaggerated upon hyperventilation. It appears evident that changes in ECM composition and consequent alteration of its mechanical and dynamic properties will affect lung function. 

The ECM is a highly dynamic complex that varies in composition according to its tissue localization and physiological environment. It has been reported that the ECM plays a crucial role in mediating the physiological function of the lung as structural support, as well as by driving proliferation, migration, EMT, and apoptosis [72]. In the human lung, ECM is mainly composed of collagen type I, elastins, laminins, and collagen IV, which have the pivotal roles of maintaining the structure, providing the elasticity, and constituting the bulk of the alveolar and airway epithelial basal lamina, respectively. It has been shown that the architecture of normal lungs presents aligned matrix fibers and a homogenous alveolar epithelial basal lamina. Conversely, IPF lungs are characterized by disorganized matrix fibers, with complete disruption of the alveolar epithelial basal lamina. Booth et al. identified a signature of 85 matrisome proteins with a different expression between normal and IPF lungs. IPF lungs are enriched in glycosaminoglycans (GAGs) and TGF-β and deficient for laminin’s α-3 (LAMA3), β-3 (LAMB3), and α-2 (LAMC2) chains, all of which are components of the alveolar epithelial basal lamina. As expected, they found collagens III (COL3A1) and VI (COL6A1) to be significantly upregulated in human IPF lungs. All these data suggest that there is a strong link between increased ECM stiffness and differentiation of fibroblasts. The activation of TGF-β in ATIIs and fibroblasts is also due to the mechanical stress deriving from a stiff ECM and could influence the tissue microenvironment and cell phenotype and function. In this respect, MMP expression is tightly regulated in the healthy lung [73]. In terms of physiological conditions, MMP activity is low but increases during repair and during pathological tissue remodeling, as in IPF. Accordingly, the lungs of patients affected by IPF showed upregulation of MMP1, 3, 7, 8, and 9 [74,75]. Interestingly, MMP1 and MMP7 are also SASP factors found to be upregulated in the lungs of patients with progressive fibrosis [50,51,54]. On the contrary, there are MMPs (MMP19 and MMP13) that showed an antifibrotic effect and are downregulated in IPF patients. The role of MMP14 is currently the subject of debate, as several studies have confirmed that it is upregulated in IPF patients [76], whereas a recent publication using a conditional mouse model of lung epithelium-specific MMP14 genetic deletion showed that a lack of MMP14 in lung epithelial cells impaired the spontaneous regeneration of the lung and increased the progression of fibrotic lesions when mice were treated with bleomycin. The absence of MMP14 resulted in increased expression of senescence-associated markers [76,77]. 

More research should be conducted to (1) elucidate the role of MMPs and SASP factors in the pathological conditioning of ECM and (2) systematically understand how different ECM compositions affect ATII and fibroblast biology.

## 3. Current Knowledge of Cellular and Molecular Mechanisms in Lung Regeneration

### 3.1. Lung Embryogenesis and Development

The lung is an organ that, due to its direct connection to the external environment, maintains an intrinsic, albeit limited, regenerative capacity [78]. Independently of the type of insult (aging, environment, genetics, or pathogens), alveolar epithelial damage usually evolves in one of two directions: (1) proper alveolar repair, leading to preserved organ function; (2) improper alveolar repair, leading to progressive fibrosing interstitial lung disease and impaired lung function. Thus, in order to gain insight into how lung regeneration occurs in the adult lung, it is important to first understand the basics of how the lung is constructed during embryonic development, where rapid proliferation and differentiation are the rule rather than the exception. The development of human lungs takes place during two main phases: (1) prenatal development (between twenty-six days and thirty-six weeks), encompassing the embryonic, pseudoglandular, canalicular, saccular, and alveolar stages; and (2) postnatal lung development, which comprises alveolarization and microvascular maturation [79]. First, the endoderm of the respiratory system is specified on the ventral side of the anterior foregut endoderm. This specification event is detected by the expression of the earliest known marker of the lung epithelial lineage, the transcription factor Nkx2.1, which will be further expressed by all lung epithelial lineages during organ development [80]. The lineage specification of the lung from endodermal foregut precursor cells is mechanistically regulated by various signaling molecules and pathways, such as sonic hedgehog (Shh) [81], retinoic acid [82], fibroblast growth factor 10 (Fgf10) [83,84], bone morphogenic protein-4 (Bmp4) [85], and Wnt/β-catenin pathway [86]. Following lineage specification, on the 26^th^ day, embryonic development of the lungs begins with the ventral outpouching of the endodermal foregut, known as the respiratory diverticulum, and its first bifurcation to form primary bronchial buds [79], which are the rudiments of the left and the right primary bronchi of the respiratory tree [80]. The branching process continues until the 36th week of embryonic development when the distal regions of the branch tips generate air sacs called alveoli [80]. The epithelial cells of the proximal and distal airways diverge early during fetal lung development and are respectively marked by the expression of the transcription factors Sox2 (proximal conducting airways) and Sox9 (distal gas-exchanging alveoli) [87]. Additional markers of the distal progenitor Sox9-positive cells are surfactant protein C (SftpC), Bmp4, and the transcription factor Id2. Next, Sox9-positive/SftpC-positive cells differentiate into ATII cells, which further differentiate into alveolar epithelial type I (ATI) cells. Using lineage-tracing studies in mice, Barkauskas et al. showed that ATIIs also constitute a pool of ATI progenitors that maintain this function even during adult life. Around the 7th month of gestation, when branching morphogenesis ceases and alveolarization begins [88], widening of the acinar airways occurs, forming the sacculi. As a result of widening, condensation of the mesenchyme occurs, and primary septa are formed at the junction of two airspaces. The surface of the primary septa is lined by ATI cells, whereas the remaining surface is filled in by ATII cells. Following birth, alveolarization continues until early adulthood, with the formation of secondary septa [80]. 

### 3.2. Alveolar Type II Pneumocytes and Their Response to Lung Injury

The critical function of gaseous exchange by the lungs is maintained by the ATI and ATII alveolar epithelial cells. ATII cells can also act as adult stem/progenitor cells, as is known to occur after lung injury, by transdifferentiating into ATI cells and promoting lung regeneration. 

Their unique ability to trans-differentiate into ATI cells allows for the physiological regeneration of the lung epithelium [89,90,91]. The mechanisms of lung regeneration are still poorly understood due to the difficulty in purifying and maintaining primary ATII cells in culture both from adult and embryonic human lungs [92]. To overcome these obstacles that have limited research on alveolar regeneration and disease, several laboratories have recently started using induced pluripotent stem cells (iPSC), differentiating them into ATII cells. In particular, Hawkins et al. revealed that iPSC, before differentiating into ATII cells, expresses high levels of NKX2.1, the earliest known marker of the lung epithelial lineage during embryogenesis [80,93]. It was shown that NKX2.1 null mutant mice presented hypoplastic lungs that are unable to mature. Next, human children with NKX2.1 mutations develop respiratory insufficiency, hypothyroidism, and neurological diseases [94]. 

Another important lung progenitor marker known to be involved in the development of the lung is SOX9. It has been shown that during embryogenesis, ATII cells arise from highly proliferative SOX9-positive cells [87]. This population represents undifferentiated progenitor cells, which progressively differentiate during development in ATII cells without contributing to the transdifferentiation from ATII to ATI cells [87]. There are no published studies about the conditions and role of SOX9-positive cells during lung injury or lung diseases.

As recently shown by Duerr et al., the conditional knockout of the ubiquitin ligase Nedd4-2 in ATII cells leads to chronic lung disease sharing key features with IPF, including progressive fibrosis and bronchiolization with increased expression of Muc5b in peripheral airways and honeycombing. This evidence further supports the concept that impaired ATII cells can drive IPF onset, on the one hand, by acting on surrounding fibroblasts via paracrine mechanisms, on the other hand, by limiting the physiological regenerative mechanism mediated by ATII to ATI transdifferentiation [95].

The process of transdifferentiation occurs at a very low rate in a normal lung. However, in response to injury, ATII cells proliferate rapidly and transdifferentiate into ATI cells to restore the normal function of the lungs [40,96]. The transdifferentiation process between ATII and ATI cells remains unclear. During development, the generation of the distal epithelium is driven by a bipotent progenitor that coexpresses both ATII and ATI markers [97]. In a recent study, Strunz et al. discovered, using a mouse model of pulmonary fibrosis, an intermediate alveolar epithelial cell marker: KRT8. Interestingly, they did not find KRT8-positive cells in the parenchyma of normal adult mouse lungs but only in the first phase of the fibrotic process. They also showed that rare KRT8-positive cells derived from ATII cells and mediated the process of transdifferentiation of ATII into ATI cells during the regenerative process. Therefore, subpopulations of ATII cells might serve as a pool of stem/progenitor cells [98,99].

It is speculated that the niche where ATII cells reside determines their fate of differentiation to ATI cells [100]. Therefore, according to Nabhan et al., the stemness of the stem cell subpopulation of ATII cells is controlled at the molecular level via Wnt signaling. This happens when ATII cells are in close contact with the niche fibroblasts that constitutively express Wnt5a [101]. Moreover, FGF signaling is required in lung development. In particular, FGF7 and FGF10 secreted from the lung fibroblasts contribute to the proliferation and differentiation of ATII cells [91]. 

**Figure 3:**
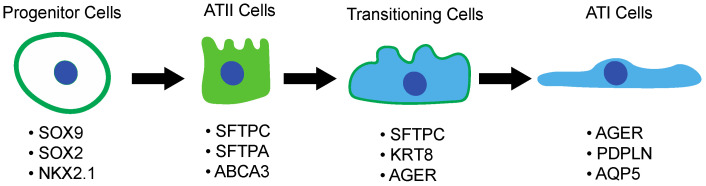
Transdifferentiation process from progenitor cells to ATI cells. Schematic depiction of the sequential differentiation process that occurs during cell differentiation into ATI cells as a response to injury, starting from a niche of SOX9/SOX2/NKX2.1-positive progenitor cells, which differentiate into ATII cells expressing SPC, SPA, and ABCA3. ATII cells then undergo a transition phase, where they begin to express KRT8 and markers of both ATII (SPC) and ATI cells (RAGE). Finally, from this intermediate state, cells transdifferentiate into terminally differentiated ATI cells positive for RAGE, podoplanin (PDPLN), and aquaporin 5 (AQP5).

Recently, a new ATII-progenitor cell population called RAS cells was discovered for the first time [100]. The RAS cells population is described as a distal lung progenitor niche that rapidly differentiates into ATII cells under the strict control of Notch and Wnt signaling pathways [100]. RAS cells (SCGB3A2+) are found in the proximal upper airway, localized in close proximity to respiratory bronchioles and show a transcriptional signature that can be categorized between canonical secretory cells and ATII cells [100]. Interestingly, the accumulation of transitioning SCGB3A2+ AT2+ cells has been observed in the lungs of both human and ferrets upon smoking exposure, as well as in the lungs of human patients with COPD, suggesting that a stall in ATII-to-ATI transitioning can impair proper lung repair and promote disease progression [100] (Figure 3). 

From this perspective, preclinical studies have involved attempts at ATII-cell based therapies to treat IPF. In particular, it has been shown that intratracheal transplantation of ATII cells can reverse lung fibrosis by effectively reducing collagen deposition and restoring SPC levels in IPF animal models [102,103]. A follow-up study in patients with moderate and progressive IPF showed the tolerability and safety of ATII intratracheal transplantation at 12 months without showing any data regarding the efficacy of the treatment in reducing lung fibrosis [104]. Currently, there is just one randomized clinical trial in phase 1 for the use of cell-based therapies against IPF, currently enrolling as many as 24 patients (NCT04262167). Therefore, this therapeutical approach is intrinsically limited by the availability of primary-cell donors and therefore impractical to scale up to the level of large, randomized clinical trials.

To overcome this issue, the use of induced pluripotent stem cells (iPSCs) as source of ATII cells can represent a viable alternative. Very recently, a study conducted by Palomo et al. indicated that iPSC-derived ATII cells, when transplanted to a bleomycin-induced IPF model, halted and reversed lung fibrosis. According to this study, inhibition of TGF-β, a known profibrotic factor, and α-SMA was observed upon transplantation. However, it is still largely unclear whether this effect was observed as a result of transplantation or due to the paracrine effect of the transplanted cells; studies regarding the fate of implanted iPSC-derived ATII cells are lacking [105].

## 4. Current Therapies for IPF 

IPF has a complex and still not fully understood pathogenesis in which the complex interplay of genetic and environmental factors is acting on multiple cell types, thus exponentially increasing the complexity of the molecular processes behind IPF onset and development. Thus, the ideal treatment for this disease should effectively target multiple pathways in order to block disease progression whilst promoting ATII-driven alveolar regeneration.

In the past, the conventional standard of care for IPF was a combination treatment with corticosteroids, Azathioprine, and a mucolytic agent (N-acetylcistein), but it was never supported by strong evidence of efficacy and was definitively stopped in 2012 upon the results of a multicenter randomized trial showing increased risks of death and hospitalization in PF-treated patients compared to the placebo group [106].

In 2014, the results of the ASCEND phase 3 clinical trial (NCT01366209) purposed pirfenidone as a novel, effective antifibrotic therapy for IPF. Pirfenidone-treated patients showed significant reduction in the risk of forced vital capacity (FVC) decline, increased 6 min walk distance (6MWD), milder dyspnea, and slower disease progression compared to the placebo group. In 2016, similar results were obtained with nintedanib (triple angiokinase inhibitor targeting FGFR, PDGFR, and VEGFR), which showed efficacy in reducing IPF progression in two phase 3 clinical trials (INPULSIS-1 (NCT01335464) and INPULSIS-2 (NCT01335477)). In 2015, all this evidence led to the update of the 2011 clinical practice guidelines through conditional recommendation of the use of nintedanib and pirfenidone for the treatment of patients with IPF [107].

Two recent phase 4 clinical trials (NCT02648048 and NCT02598193) showed both the safety and the improved efficacy of combination therapy with nintedanib and pirfenidone [108,109]. It was recently shown that the response to antifibrotic treatment is also effective in patients with genetic polymorphisms in IPF-associated genes (MUC5B T* allele or DSP G* allele) [110]. However, it is important to remember that these drugs do not cure the disease but only slow down its progression, which is why it is fundamental to establish novel therapeutic approaches, including combination therapies and, eventually, therapies aimed at not only blocking fibrosis but also promoting ATII-driven alveolar regeneration.

The scientific community is tirelessly attempting to dissect the complex molecular mechanisms sustaining the pathogenesis of IPF, and thanks to this effort, new potential druggable targets are continuously being identified and explored in early clinical trials. Given the latest scientific work supporting the relevance of cellular senescence in the onset of IPF (see above), an innovative approach based on the selective ablation of senescent cells using senolytics (dasatinib plus quercetin) has been shown to alleviate IPF-related dysfunction in bleomycin-administered mice [50]. A recent open-label pilot study confirmed this evidence in humans in a small cohort of IPF patients (26), although the efficacy of senolytics for IPF therapy still has to be proven in lager randomized trials [69].

At present, there are 16 interventional clinical trials actively enrolling for IPF treatment, of which the majority are phase 1 (7) and phase 2 (5) studies for new molecules (Table 1). 

Among these studies, there are two large phase 3, randomized, double-blind, placebo-controlled efficacy and safety studies to test PRM-151 (NCT04552899) and FG-3019 (NCT03955146) on 658 and 340 IPF patients, respectively.

PRM-151 is the human recombinant of pentraxin 2 (PTX-2), a circulating protein that binds to monocytes and inhibits their differentiation into profibrotic fibrocytes and TGF-β-producing macrophages [111]. PTX-2 levels have been found to be decreased in patients with IPF [112], and administration of PRM-151 inhibited fibrosis development in the bleomycin mouse model of lung fibrosis [113]. In 2018, a phase II study showed an acceptable safety profile for PTX-2, demonstrating a significant effect on reducing pulmonary functional decline over 24 weeks [114].

FG-3019 (pamrevlumab) is a fully recombinant human monoclonal antibody anti-CTGF (connective tissue growth factor). CTGF has been shown to play a central role in the process of fibrosis by upregulating the TGF-β pathway [115]. Accordingly, elevated CTGF levels were found in plasma samples of IPF patients [116]. In 2017, the results of the phase 2, randomized, double-blind, placebo-controlled trial PRAISE showed a significant effect on reducing pulmonary functional decline with an acceptable safety profile in 160 patients with IPF. A phase 3, randomized, double-blind, placebo-controlled, multicenter trial is currently enrolling to validate pamrevlumab efficacy in a large cohort of IPF patients (340) with a primary endpoint of reduced FVC at 48 weeks (NCT03955146). 

Clearly, aiming at the development of new proregenerative therapies for the lung is an ambitious goal. To achieve proper alveolar regeneration and, eventually, neoalveologenesis, multiple cellular players need to be finely orchestrated to co-operate in this complex biological process. To this end, a considerable scientific effort must be put in place to:

(1) Obtain better mechanistic insights into the interplay between ATII cells and fibroblasts during fibrosis progression, with the aim of identifying novel ATII-related druggable targets for the development of the most effective antifibrotic therapies;
(2) Systematically dissect the molecular mechanisms of ATII-driven physiological lung regeneration in order to find new druggable targets for the development of effective regenerative therapies;
(3) Tackle this disease by simultaneously blocking fibrosis progression and promoting alveolar repair and organ regeneration.

**Figure 4:**
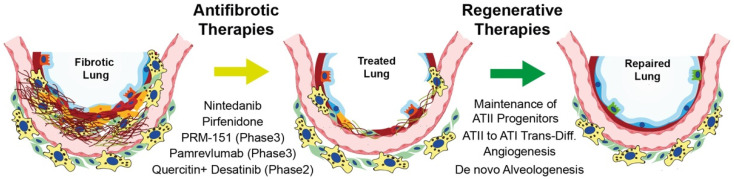
Novel therapeutic strategies for IPF treatment. Summary illustration of putative strategies to (1) stop fibrosis progression by hampering aberrant ECM deposition and eliminating defective cells (antifibrotic and senolytic drugs) and (2) bring back the lung to a healthy condition by promoting alveolar regeneration (regenerative therapies).

For this reason, therapeutical strategies should be aimed not only at stabilizing or slowing down the disease and improving quality of life but also at reverting the disease by discovering new effective therapies that promote alveolar regeneration (Figure 4). 

## Figures and Tables

**Table 1:** Summary of all the interventional clinical trials for IPF that are actively enrolling patients.

Treatment	Estimated Enrolled Patients	Type	Clinical Trial ID
Pamrevlumab (FG-3019)	340	Phase 3	NCT03955146
PRM-151	700	Phase 3	NCT04594707–NCT04552899
Treprostinil	396	Phase 3	NCT04708782
ENV-101	60	Phase 2	NCT04968574
PLN_74809	84	Phase 2	NCT04396756
GKT137831	60	Phase 2	NCT03865927
GB0139	500	Phase 2	NCT03832946
C21	60	Phase 2	NCT04533022
Jaktinib Dihydrochloride Monohydrate	90	Phase 2	NCT04312594
Saracatinab	100	Phase 1	NCT04598919
ZSP1603	36	Phase 1	NCT05119972
INS018_055	80	Phase 1	NCT05154240
AZD5055	104	Phase 1	NCT05134727
SHR-1906	60	Phase 1	NCT04986540
NIP292	72	Phase 1	NCT04720443
Inhaled Nitric Oxide (iNO)	40	Early Phase 1	NCT05052229
